# AlloPipe and Its Web Server Allogenomics: From Genomic Data to Candidate Minor Histocompatibility Antigens

**DOI:** 10.1111/tan.70590

**Published:** 2026-02-11

**Authors:** Adèle Dhuyser, Pierre Delaugère, Pierre Laville, Robert Clerc, Alice Aarnink, Laurent Mesnard, Hugues Richard

**Affiliations:** ^1^ HLA and Histocompatibility Laboratory CHRU de Nancy Vandoeuvre‐les‐Nancy France; ^2^ IMoPA, UMR7365 CNRS, Université de Lorraine Vandoeuvre‐les‐Nancy France; ^3^ Institute of Computing and Data Sciences (ISCD), Sorbonne Université Paris France; ^4^ Sorbonne Université Maison des Modélisations Ingénieries et Technologies (SUMMIT), Sorbonne Université Paris France; ^5^ CoRaKiD, INSERM UMR1155, Sorbonne Université Paris France; ^6^ Eurofins Biomnis Laboratory Lyon France; ^7^ Soins Intensifs Néphrologiques et Rein Aigu (SINRA), Hôpital Tenon, Assistance Publique des Hôpitaux de Paris Paris France; ^8^ Bioinformatics and Translational Research Team – Genome Competence Center (MF1), Robert Koch Institut Berlin Germany; ^9^ Laboratory of Computational and Quantitative Biology (LCQB), UMR 7238 CNRS Sorbonne Université Paris France

**Keywords:** bioinformatics, genomics, minor histocompatibility antigens, transplantation, web interface

## Abstract

While transplantation outcomes are closely linked to HLA compatibility, minor histocompatibility antigens (mHAgs)—peptides embedded in HLA molecules bearing amino acid differences within the donor/recipient pair—also contribute to T‐cell alloreactivity, especially in HLA‐matched pairs. Because mHAgs initially arise from non‐synonymous genomic polymorphisms, we and others have focused on genomic data to approximate mHAgs load. However, most teams based their strategies on genotyping data, which only allows the study of polymorphisms that are already known and well described at the population level. Nowadays, no publicly available software exists that can facilitate unbiased identification of mHAgs candidates from whole‐exome or whole‐genome sequencing data. We present AlloPipe, a bioinformatics tool designed for this purpose. AlloPipe operates in two sequential steps: Allo‐Count, which accurately identifies directional amino acid mismatches, and Allo‐Affinity, which reconstructs peptides around these mismatches and returns their affinity for HLA class I molecules. With its ability to process rapidly large datasets in modest‐scale computing infrastructures in a clinically relevant timeframe, AlloPipe makes possible the implementation of class I restricted mHAgs approximation into clinical decisions, such as immunosuppressive therapy optimization or donor selection. This open‐source software offers a flexible choice of tools at each step of the analysis, especially when predicting affinity towards HLA molecules. It is available locally and via a web interface, making potential mHAgs predictions more accessible and actionable in clinical practice.

## 1 Introduction

### 1.1 Alloreactivity and Transplantation

Alloreactivity refers to the activation of specific immunity following an immunising event—pregnancy, transfusion, or transplantation. The activation of T‐lymphocytes through direct and indirect recognition processes is the cornerstone of the initiation of alloreactivity. It requires the recognition of an (HLA‐peptide) complex that is different from the self. The HLA molecule and/or its embedded peptide carry this difference [1].

After solid organ transplantation (SOT), recipient‐driven alloresponses lead to the HvG effect (host versus graft) potentially driving clinical rejection. The recipient's T‐lymphocytes—after having undergone negative and positive selection upon contact with [HLA_recipient_‐peptide_recipient_] complexes—may first encounter the donor's dendritic cells from the graft that present [HLA_donor_‐peptide_donor/recipient_] complexes in the context of direct alloreactivity. Then, as the graft dendritic cells are depleted and the recipient dendritic cells re‐colonise the graft, the recipient T‐lymphocytes will encounter the [HLA_recipient_‐peptide_donor/recipient_] complexes as part of indirect alloreactivity.

Allogeneic haematopoietic cell transplantation (allo‐HCT) usually results in complete lymphodepletion in the recipient, so donor‐driven alloresponses occur, responsible for a GvH effect (Graft‐versus‐Host) and/or a GvL effect (graft‐versus‐leukaemia). The donor's T‐lymphocytes—having been negatively and positively selected upon contact with the [HLA_donor_‐peptide_donor_] complexes—may first encounter the recipient's dendritic cells, which have [HLA_recipient_‐peptide_donor/recipient_] complexes as part of direct alloreactivity. Then, as the recipient's dendritic cells are depleted and replaced by the donor's dendritic cells, the donor T‐lymphocytes will encounter the [HLA_donor_‐peptide_donor/recipient_] complexes as part of indirect alloreactivity. However, in complex settings like mixed chimerism, recipient‐driven alloresponses might also happen, leading to HvG and potentially graft rejection, also after allo‐HCT.

### 1.2 Minor Histocompatibility Antigens (mHAgs)

Transplants from intra‐familial HLA‐identical donors—which are assumed to have the same entire HLA regions—can still lead to clinically significant HvG after SOT [2] or GvH after allo‐HCT, sometimes even leading to severe GVHD [3]. This indicates that other compatibility systems are involved in these alloreactivity processes. The KIR system and the MIC‐A/MIC‐B polymorphisms have been particularly studied to support additional NK cells' role in the alloreactivity [4, 5]. However, sticking to the T‐lymphocyte mediated alloreactivity, only differences in HLA embedded peptides can explain lymphocyte activation given that donor/recipient HLA molecules are indeed identical.

Those embedded peptides are called mHAgs, that is, defined by (i) a genetic polymorphism between donor and recipient, (ii) responsible for an amino acid variation in an expressed protein, and (iii) capable of being embedded into the HLA molecule groove [6]. As there are ubiquitously expressed proteins and others whose expression is restricted to certain tissues, mHAgs are either ubiquitous or organ specific, the seconds therefore called tissue‐related antigens [7].

mHAgs embedded in HLA class I molecules generally derive from the degradation of intra‐cytoplasmic proteins; they are then presented to the TCRs of CD8+ T‐lymphocytes, whose activation is responsible for cytotoxicity towards the mHAgs presenting cell. Thus, after SOT, the amount of mHAgs embedded in HLA class I expressed in the transplanted organ should allow approximation of organ‐specific alloreactivity and correlate with recipient‐driven responses, thus HvG potential and graft rejection risk. After allo‐HCT, mHAgs restricted to haematopoietic tissues hold the promise of a donor‐driven response leading to a GvL effect without triggering GvH effect.

### 1.3 Identification of mHAgs

Historically, mHAgs have been identified from alloreactive lymphocyte clones after transplantation, either by elution of peptides embedded in HLA class I molecules prior to identification by mass spectrometry [8]. Studies have then reported extensively on the benefits of genotyping using DNA microarrays covering more single‐nucleotide polymorphisms (SNPs), and Armistead et al. provided a proof of concept that mHAgs can be predicted using bioinformatics [9]. Martin et al. assessed a *‘genome‐wide mismatch’* of over 19,000 SNPs within donor/recipient pairs for allo‐HCT [10], while Reindl‐Schwaighofer et al. processed nearly 60,000 SNPs within kidney transplant pairs [11]. In both cases, the number of SNPs mismatches within the pair correlated with the adverse effects of alloreactivity, that is, GvH and HvG effects, respectively. However, DNA microarrays only allow the study of polymorphisms that are already known and well described, which might be responsible for a signal distortion.

Therefore, we and others performed whole‐exome sequencing (WES) as an unbiased approach for SNPs enumeration within a donor/recipient pair. Mesnard et al. reported the correlation between non‐synonymous directional mismatches and chronic graft rejection after kidney transplantation [12]. More recently, Cieri et al. successfully reported the impact of mHAgs identified from WES while integrating organ‐specific transcriptomes, single‐cell resources and proteome expression [13]. However, despite the demonstrated importance of mHAgs prediction from transplant pairs, there are currently no publicly available tools that can accurately and reliably predict mHAgs candidates.

**FIGURE 1:**
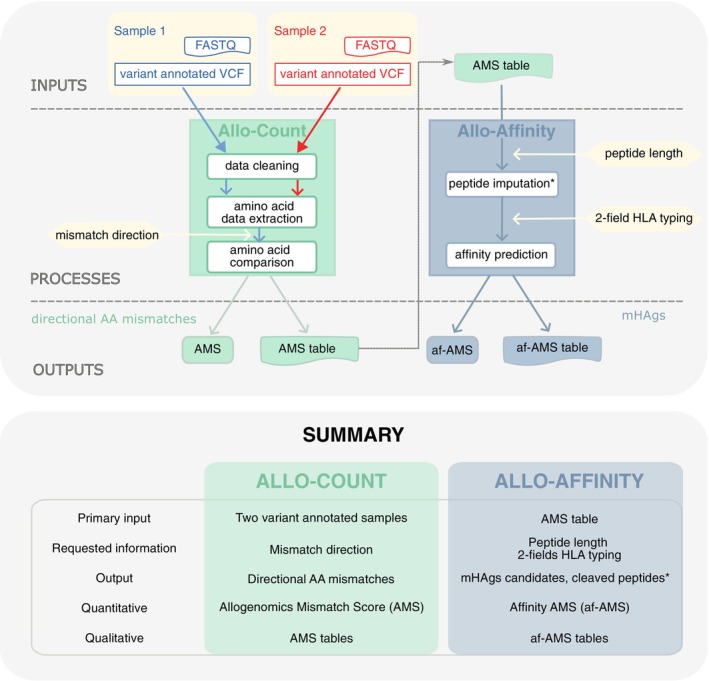
Global overview of the AlloPipe tool. Top panel: The AlloPipe tool runs from the VCF variant annotated file from each sample. Allo‐Count first performs a stringent data cleaning, and then compares the amino acid sequence of both samples in the direction of interest. Allo‐Count returns the number of directional amino acid mismatches as the Allogenomics Mismatch Score—AMS—with qualitative information in the AMS tables. Allo‐Affinity takes the AMS tables as input and then reconstructs the set of peptides that are different between the two samples using a sliding window of the specified peptide length. In parallel, cleavage sites and the resulting cleaved peptides overlapping mismatches can be predicted using NetChop (depending on sample genotype and on directionality, see methods). The affinity of each of the resulting peptides for the given HLA molecules is then calculated by NetMHCpan 4.1 to generate the ‘Affinity AMS’ (af‐AMS) table that regroups the imputed peptides and their affinity towards the HLA molecules. From this table, the user can set an affinity threshold to decide which peptides are considered as mHAgs and which will contribute to the af‐AMS. Bottom panel: Table summarising the inputs and outputs of each AlloPipe module.

The AlloPipe tool presented here makes the best use of data and resources readily available in medical routine, that is, whole exome sequencing, to predict a group of mHAgs candidates. AlloPipe is divided into two sequential parts, Allo‐Count and Allo‐Affinity (Figure 1). After a first step of stringent data cleaning to ensure sufficient control over the called genotypes, Allo‐Count returns the directional amino acid mismatches extracted from D/R exome‐to‐exome comparison in a matter of minutes. Allo‐Affinity then returns the mHAgs candidates for a given HLA class I typing—based on NetMHCpan 4.1 predictions by default [14]—within a few hours. Prediction of cleaved peptides is also possible based on NetChop [15]. At each stage of the pipeline, AlloPipe provides both quantitative and qualitative insight into the compatibility profile between the donor and the recipient. The allogenomics.com web server additionally enables predictions to be more accessible in clinical practice, where bioinformatics support is limited.

## 2 Material and Methods

### 2.1 The AlloPipe Tool

AlloPipe is a bioinformatics tool to infer candidate mHAgs from two whole‐exome sequenced samples. Nevertheless, large gene panels or whole genome sequencing can also serve as an input. AlloPipe is divided into two sequential steps and modules, Allo‐Count and Allo‐Affinity, which provide insight into the directional amino acid mismatches and the candidate mHAgs expected from the pair of samples, respectively (Figure 1, upper panel).

AlloPipe takes as input two genotype files saved in Variant Calling Format (VCF). The files must also contain information about the expected amino acid changes at positions with non‐synonymous polymorphisms. Such a file can be generated using the Variant Effect Predictor (VEP) tool via the command line or the web interface [16]. By default, Allo‐Count expects amino acid changes to be annotated by VEP, but it is possible to adapt the output coming from another variant predictor.

**FIGURE 2:**
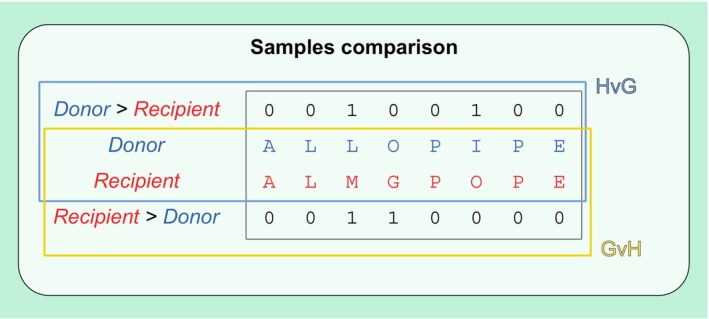
Mismatch directionality. Allo‐Count compares the donor's and the recipient's information about the amino acid changes expected at positions with non‐synonymous polymorphisms. The sample comparison is directional and accounts for either the amino acids that are present in the donor but absent in the recipient (mHAgs of interest after solid organ transplantation) or that are present in the recipient but absent in the donor (mHAgs of interest after haematopoietic stem cell transplantation).

Allo‐Count first performs a stringent data cleaning step, the parameters of which can be defined by the user (Supporting Information: Appendix 1). The extraction of information on the retained variant allows for a directional comparison of the samples (Figure 2). Finally, Allo‐Count returns the Allogenomics Mismatch Score (AMS), which is a discrete quantitative variable measuring the directional amino acid mismatches reflecting the alloreactivity potential within a donor/recipient pair. It also provides an AMS table that details each of the mismatches contributing to this score, pinpointing candidate mHAgs (Supporting Information: Appendix 2 details the table format).

The Allo‐Affinity module takes the AMS table as input. Peptides of the required length are reconstructed around each previously detected amino acid change using a sliding window. The affinity of these peptides towards the HLA molecules present in the pair is obtained using NetMHCpan‐4.1 for the HLA class I molecules [14]—although other affinity predictors might be used such as MixMHCpred [17]. Allo‐Affinity returns a summary af‐AMS table: the reconstructed peptides are reported together with their affinity scores and their related information such as the originating transcript or its genomic coordinates (see Supporting Information: Appendix 3).

If donor and recipient have been genotyped separately, obtaining a joint genotype has to deal with missing information: a position that contains a variant doesn't necessarily have an explicit genotype in the other pair. Two behaviours are encoded in Allo‐count to treat those cases: (1) stringent (default): those positions are masked and not counted. (2) Imputation: positions whose genotype is missing are treated as reference.

Allo‐Affinity also integrates an option to predict the cleavage sites and resulting cleaved peptides using NetChop [15]. Returned cleaved peptides can then be used in an additional filtering of the peptides reported by Allo‐Affinity. This prediction works in two steps: (i) reconstruction of the protein sequence using the genotype of the sample producing potential mHAgs (donor for SOT, recipient for allo‐HCT), (ii) prediction of the cleavage site on proteins containing directional amino acid mismatches using NetChop [15].

AlloPipe therefore produces four main outputs for each donor/recipient pair consisting of two scores (AMS and af‐AMS) together with their corresponding summary tables (Figure 1, bottom panel and Appendices 2 and 3 for more details about the tables).

### 2.2 The Allogenomics Web Server

The allogenomics.com web server performs the same steps as the AlloPipe pipeline within a user‐friendly web interface. The affinity prediction tools cannot be hosted directly on the web server due to their licensing conditions, but the peptides list returned by Allo‐Affinity can easily be processed online by one of the prediction tools afterwards. This allows more flexibility in the choice of the affinity predictor (such as for instance NetMHCpan 4.1 [14], or MixMHCpred [17]).

The web server offers additional functionalities such as: (i) restriction to genomic regions of interest by specifying a BED (Browser Extensible Data) file, (ii) restriction to a list of rsIDs from dbSNP—that is, to retrieve the described mHAgs, (iii) restriction to transcript IDs—that is, to focus on tissue‐restricted mHAgs and (iv) restriction to predefined lists of transcripts expressed in various organs (such as kidney or bone marrow). Those lists are compiled using expression data from the Protein Atlas database [18]. Finally, the distribution of the AMS calculated for related and unrelated pairs over our three cohorts is provided to the user to ease interpretation.

### 2.3 Available Data and Exome Sequencing

To evaluate our tool, we performed the whole exome sequencing in three different cohorts of allo‐HCT pairs. One of 48 related HLA‐matched pairs (Cohort 1), then 23 additional related HLA‐matched pairs (Cohort 2), and 40 additional related semi‐HLA‐matched pairs (Cohort 3). For cohorts 1 and 2, exons were captured using the Human Twist Exome Refseq 40 Mb (Twist Biosciences, San Francisco, CA), followed by 150‐bp paired‐end sequencing performed on Ilumina NovaSeq 6000 instrument (Illumina Inc., San Diego, CA). For cohort 3, exons were captured using the Human Twist Comprehensive Exome 42.2 Mb (Twist Biosciences, San Francisco, CA) followed by 100‐bp paired‐end sequencing performed on Illumina NextSeq 2000 instrument (Illumina Inc., San Diego, CA). In cohort 2, we also added one patient from the two previous cohorts to assess inter‐platform variability (with Cohort 3) and batch effect or the impact of a potential change in sequencing practice over time (with Cohort 1). Details of the secondary analyses are provided in Supplementary Data 1 and 2. All patients gave their written consent for the generation and use of genetic data for research purposes, as well as clinical data collection. We also created a fourth artificial cohort in which we created artificial pairs by sampling unrelated donor and recipient pairs within each of the previous cohorts, resulting in a total of 4322 pairs.

### 2.4 Simulation of Donor/Recipient Pairs

To validate the accuracy of the AMS scores, we simulated data from artificial siblings using genomes from the 1000G project (family trio NA12878_1463, [19]). We performed the simulation in two steps. We first simulated genotypes of chromosome 8 in 15 siblings from the parents in the trio using the sim1000G tool [20]. Then, we used each sibling's genotype to simulate exome sequencing data using the NEAT tool [21].

### 2.5 AMS Generation

AMS were first calculated for the 111 pairs using the tool's default quality parameters (see Supporting Information: Appendix 1). In order to assess the contribution of rare variants, we set the allele frequency threshold to 0% (given the gnomAD frequencies). We also created a fourth artificial cohort in which we created artificial pairs by sampling unrelated donor and recipient pairs from the previous cohorts. For each unrelated artificial pair, we then calculated and recorded their AMS.

For the simulation, we computed the AMS in two ways for each of the possible siblings' pairs: the *theoretical AMS* obtained from the genotypes of step 1, and the *observed AMS* obtained from aligning and genotyping the individuals from the simulated reads. We did not evaluate the accuracy of the af‐AMS as it relies solely on the affinity prediction tool chosen.

### 2.6 Statistics

Normality of the AMS distribution was assessed for each cohort using the Shapiro–Wilk test with a risk value set at *α* = 0.05. When the data followed a normal distribution, a one‐way analysis of variance (ANOVA) was performed to compare the means across the groups. Alternatively, the Kruskal–Wallis test was employed when the distributions did not meet the assumption of normality. To test the equality of two distributions we performed a Kolmogorov–Smirnov test. Statistical analyses were performed with the Python programming language using the scikit‐learn and pandas modules, and the *R* statistical software.

## 3 Results

### 3.1 Description of Cohorts

The parameters of interest are limited to the degree of parenthood and the sex mismatch of the pairs. HLA high‐resolution typing was available for all individuals at the loci HLA‐A, ‐B, ‐C, DRB1 and ‐DQB1. While related HLA‐matched individuals can only be siblings, different degrees of relatedness can be found in semi‐identical cohorts (Table 1). In most cases, semi‐identical donors are children or siblings of the recipient (42.5% and 50% respectively), while other pairings—such as parents or cousins—are rare. The proportion of sex mismatch, which has been described as unfavourable (female‐to‐male) for chronic graft‐versus‐host disease (cGVHD), represented a minority of patients (15.3%), especially in the semi‐identical population, reflecting current donor selection recommendations.

**TABLE 1:** Cohorts' characteristics.

	Cohort 1 (*n* = 48 pairs)	Cohort 2 (*n* = 23 pairs)	Cohort 3 (*n* = 40 pairs)	Total
Relatedness				
Siblings	48 (100%)	23 (100%)	20 (50%)	91 (82%)
Parent‐to‐child			1 (2.5%)	1 (0.9%)
Child‐to‐parent			17 (42.5%)	17 (15.3%)
Cousins			2 (5%)	2 (1.8%)
Sex‐mismatch				
Female‐to‐male	9 (18.7%)	6 (26.1%)	2 (5%)	17 (15.3%)
Others	39 (81.3%)	17 (73.9%)	38 (95%)	94 (84.7%)

*Note:* The statistical characteristics such as relatedness and female‐to‐male ratio are listed for each of the three cohorts.

### 3.2 Variant Features Annotated by Variant Effect Predictor (VEP)

Variant annotation with VEP in our cohorts allows us to characterise their nature (Table 2). First, SNPs are the most frequently detected type of variation: 94.1%, 90.1% and 93.4% in cohorts 1, 2 and 3, respectively. Second, the majority of variants are consistently divided into missense and synonymous variants across the three cohorts. Third, there is a significant proportion of novel variants in each cohort: 3.8%, 10.9% and 6.4% respectively.

**TABLE 2:** Summary of variant features annotated by VEP.

	Cohort 1 (*n* = 96)	Cohort 2 (*n* = 48)	Cohort 3 (*n* = 80)
Total variants	152.726	109.005	125.594
Novel/existing variant			
Novel variants	**5,804 (3.8%)**	**11,858 (10.9%)**	**8,096 (6.4%)**
Existing variants	146,922 (96.2%)	97,147 (89.1%)	117,498 (93.6%)
Variant classes			
SNP	**143,694 (94.1%)**	**98,160 (90.1%)**	**117,291 (93.4%)**
Deletion	4,045 (2.6%)	5,245 (4.8%)	3,759 (3%)
Insertion	2,496 (1.6%)	4,256 (3.9%)	2,851 (2.3%)
Sequence alteration	2,223 (1.5%)	1,186 (1.1%)	1,498 (1.2%)
Indel	268 (0.2%)	158 (0.1%)	195 (0.2%)
Consequences			
Total consequences	144.147	102.349	119.3
Missense variants	**71,203 (49.4%)**	**48,411 (47.3%)**	**59,531 (49.9%)**
Synonymous variants	**60,888 (42.3%)**	**41,128 (40.2%)**	**48,433 (40.6%)**
Stop gained	1,070 (0.7%)	779 (0.8%)	1,084 (0.9%)
Others	10,986 (7.6%)	12031 (11.76%)	10,252 (8.6%)

*Note:* For each of the three cohort, the properties of the variant annotated with the variant effect predictor tool are listed.

Taken together, these figures indicate that in our cohorts, a tool that would concentrate on processing SNPs would capture at least 90% of the genetic variation of the exome. Additionally, the proportion of rare variants can reach up to 10% of the total number of variants, and this in turn could contribute to a significant proportion of the AMS within a D/R pair (see AMS distribution).

### 3.3 AMS Distribution

**FIGURE 3:**
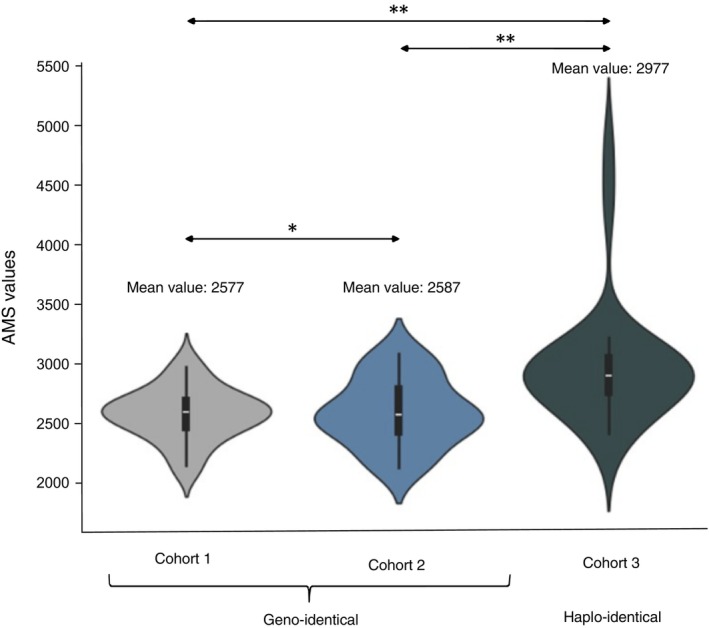
AMS distribution across the three related cohorts. Comparison of the AMS distribution across cohorts 1 through 3. ** indicates a significant difference (level 0.05) between two distributions (using the appropriate test, see methods).

**FIGURE 4:**
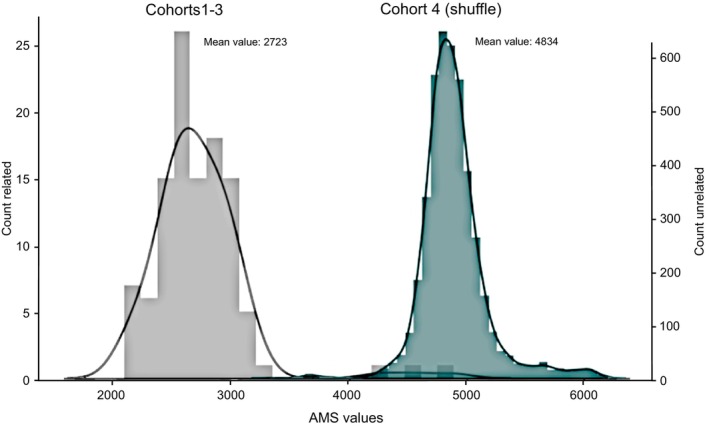
AMS distribution for related and unrelated pairs. Distribution of the AMS values for the union of cohorts 1 through 3 (related) and for cohort 4 (unrelated), obtained by considering all possible donor/recipient pairs obtained by randomly drawing pairs of samples from the cohorts (unrelated).

The mean value of the AMS is 2577 in Cohort 1 and 2587 in Cohort 2—both HLA‐identical—with no significant difference (Figure 3). The mean value of the AMS in Cohort 3—semi‐identical—is higher but includes three outlier values corresponding to the two pairs of cousins and one child‐to‐parent pair. After the exclusion of the aforementioned outlier pairs, a comparable distribution was observed between the HLA‐identical and the semi‐identical pairs (Supplementary Figure 1). The AMS values are significantly higher in the simulated pairs of unrelated D/R couples, with a mean value of 4834 (mean value of 2723 in the related pairs, Figure 4). This highlights the greater genetic difference between pairs of unrelated individuals.

**FIGURE 5:**
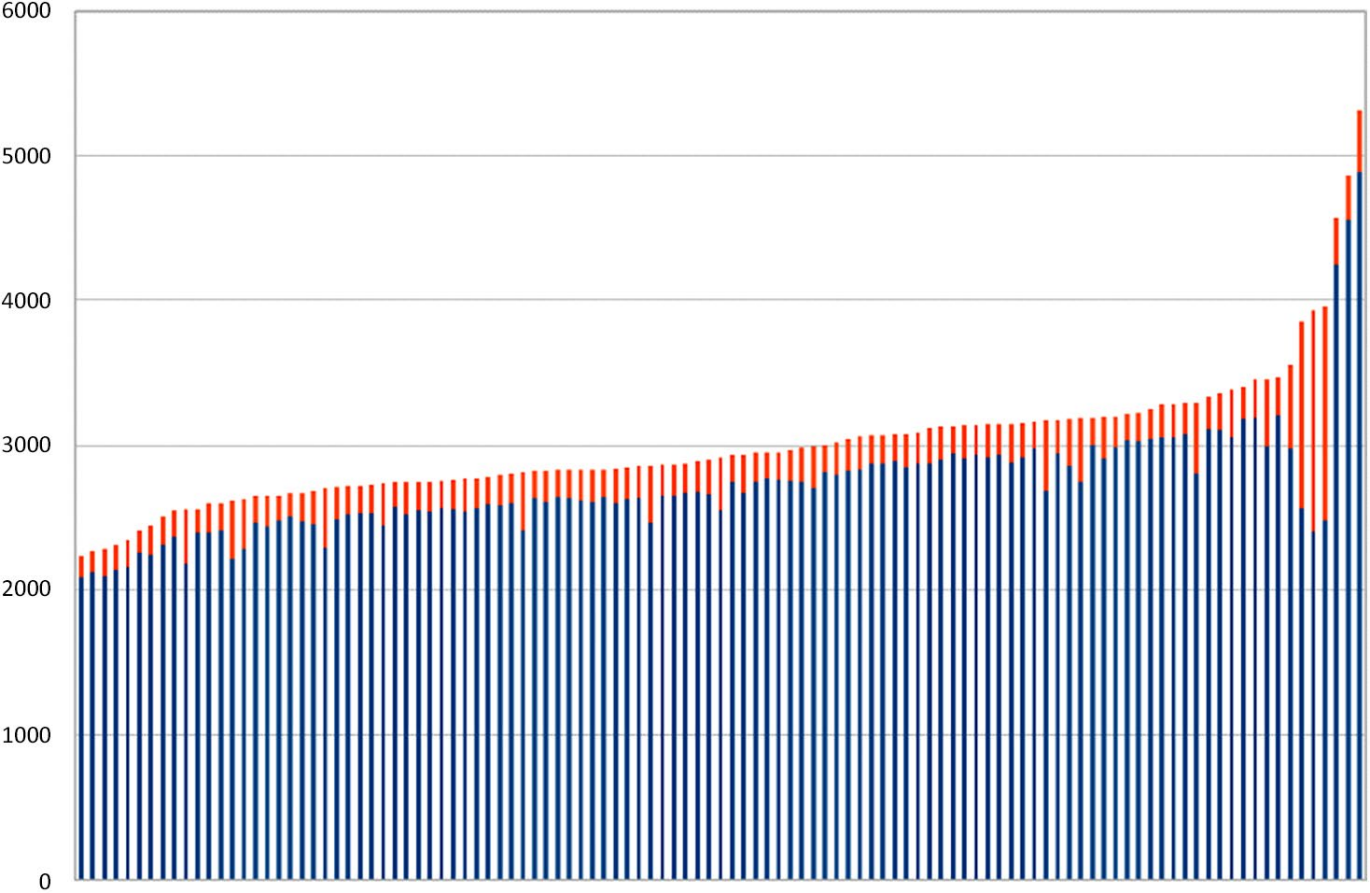
Contribution of the rare variants to the AMS. AMS values for all pairs in the three cohorts, sorted in ascending order. Blue bars: AMS values obtained when fixing the minimal gnomAD exome allele frequency at 1% (default parameter). Red bars: Change in the AMS value when the gnomAD exome allele frequency threshold is set to 0%. This corresponds to the contribution of the rare variants.

Moreover, rare variations (gnomAD allele frequency ≤ 0.01) represent a proportion of the AMS estimated at an average of 8% of the score (Figure 5, Supplementary Figure 2).

### 3.4 Technical Validation of the AMS Computation

We performed simulations to ensure that the AMS values returned by the AlloPipe tool are accurate and reflect the actual number of amino acid mismatches between two individuals. We simulated the genotype of siblings in a family from parents genotyped in the 1000G project, and then compared the theoretical AMS value with the one obtained after sequencing the exomes of the individuals (limited to chromosome 8, see methods). We observed an almost perfect correlation (Pearson *r* > 0.999) between the theoretical AMS values and the observed ones (computed based on the sequencing data) (Supplementary Figure 3a). Further, the relative difference between theoretical and observed AMS is below 2% in more than 80% of the cases (Supplementary Figure 3b). As an additional validation, we also verified that the values we obtained from the cohorts 1 and 2 when limited to chromosome 8 of related individuals are in the same range as the simulated ones. The distributions are comparable (Supplementary Figure 3c, Kolmogorov–Smirnov test not significant, *p* value = 0.088). Taken together, this validates that AlloPipe is an accurate tool to compute AMS values, and that those values are within the expected range of variation in the population.

### 3.5 Recovering Previously Described mHAgs

**FIGURE 6:**
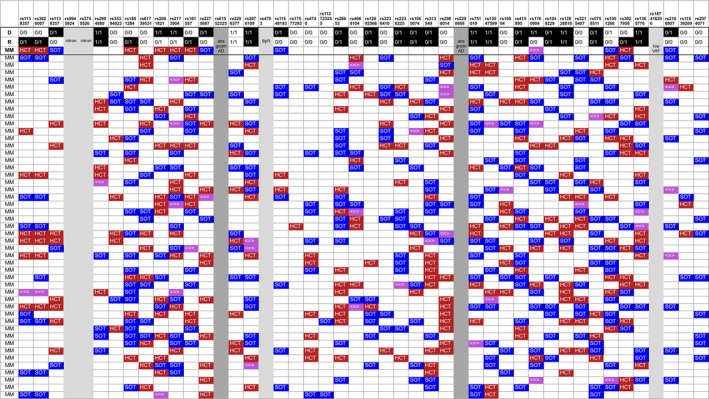
Agreement between the mHAgs described in the literature and the peptides in cohort 1. For each D/R pair of the first cohort (*n* = 47), the mHAgs listed in Oostvogels et al. [7] are highlighted in colour when observed. The colour of the box depends on the directionality of the mHAg annotated by AlloPipe‐Count: Blue for SOT (present in the donor but absent in the recipient), red for allo‐HCT (present in the recipient but absent in the donor) and pink if the mHAg was annotated in both directions. Light grey indicates mHAgs that do not alter the coding sequence. Dark grey indicates the variants not present in the gnomAD database (2 cases).

To evaluate the detection potential of AlloPipe, we verified that we could retrieve the mHAgs described in the literature. Based on the list provided by Oostvogels et al. [7], we listed the rsID corresponding to 46 mHAgs and screened them in Cohort 1 (Figure 6). For each mHAg, we were able to find at least one pair exhibiting it, except for the following five variants: rs9945924, rs3745526, rs4703, rs187416296, rs61552325, rs2298668. However, rs9945924 and rs3745526 are non‐coding variants, rs4703 is a synonymous variant, and our tool does not aim to catch those signals. Rs187416296 is a rare missense variant (AF = 0.0006) making its observation in our cohort unlikely. Finally, rs61552325 and rs2298668 are not even present in gnomAD v4.1. We note that the variant peptides previously described as mHAgs are also present in the peptide lists returned by Allo‐Affinity for each pair.

### 3.6 Accessibility and Computation Time

The source code of AlloPipe is available at https://github.com/huguesrichard/Allopipe under the MIT licence. We optimised the computation time of the tool such that it returns a score from a pair of variant annotated exomes within minutes. The expected distribution for related and unrelated donor/recipient pairs is provided on our website and on the web server.

## 4 Discussion

T‐lymphocytes allorecognition is a cornerstone of post‐transplant alloreactivity. Even in fully HLA‐matched donor/recipient pairs, HvG responsible for acute or chronic rejection can occur after SOT. Similarly, during HLA‐identical allo‐HCT GvH effect potentially leading to graft‐versus‐host‐disease (GVHD) can occur. We and others have previously validated the interest of mHAgs—peptides derived from any expressed proteins secondary embedded in HLA molecules that are different between donor and recipient, to gain insight into the non‐HLA compatibility landscape [12, 13].

Any genomic non‐synonymous polymorphisms can potentially lead to a relevant mHAg. The frequency of the polymorphism accounting for the load of mHAgs is not indicative of its dangerousness and should be scrutinised only in regard to the donor/recipient compatibility. Therefore, genotyping might not be sufficient, but instead exhaustive mHAgs inference for a particular donor/recipient pair ideally requires (i) robust exome data, (ii) unambiguous HLA typing at the allelic level and (iii) powerful and robust bioinformatics tools to combine these data. The first two points have been successfully addressed during the past years in medical laboratories. Other teams have successfully addressed the third point and highlighted the complexity of mHAgs prediction from genomic data [13]. However, they seem incompatible with clinical use and do not provide an open‐source code, nor are the associated datasets and resources available.

We have previously demonstrated the efficiency of the AMS as a conceptual approach to describe mHAgs that correlate with long‐term graft function after kidney transplantation [12]. Unlike most studies done in the field, our genomic mismatches are unbiasedly assessed from whole‐exome sequencing (WES) data and not from DNA microarray genotyping, allowing for a more exhaustive detection of polymorphisms and rare variants in coding regions [12], which can account for up to 10% of the score in our evaluation. The computing time and necessary resources are compatible with clinical use.

We have further developed the tool by adding new functionalities, so the user can: (i) address directionality from SOT to haematopoietic stem cell transplantation, (ii) use an imputation mode for missing genomic data, (iii) input data as a joint VCF file containing multiple donor/recipient pairs allowing the scores to be processed as a cohort, (iv) easily restrict the score to genomic regions, variants, genes or transcripts of particular interest, (v) reconstruct peptides of any length and (vi) retrieve the affinity of the latter peptides towards the HLA groove to sort the best candidates by their affinity, and optionally retain the most probable accounting for proteasomal cleavage. Computation time has been dramatically reduced since the last version, and an AMS is returned within minutes. The directional mismatches and peptide generation are also provided as a user‐friendly web interface. The peptide list can be processed ‘as is’ by any affinity prediction tool.

Allopipe offers a versatile framework that can be adapted to the user's needs. In the case of semi‐identical transplantation, the donor's T lymphocytes might only recognise peptides presented by shared HLA alleles. Peptides restricted to recipient‐only HLA alleles are unlikely to be immunogenic. Users can consider this hypothesis by restricting the computation of the af‐AMS to the shared haplotype. Additionally, the option to restrict the AMS and af‐AMS score to a list of transcripts expressed in specific cellular subsets can help refine the score towards GvL or GvH effect. Indeed, Graft‐versus‐leukaemia (GvL) effects are primarily driven by peptides derived from haematopoietic mHAgs, whereas graft‐versus‐host (GvH) reactions result from mHAgs expressed in non‐haematopoietic tissues such as the skin, gut and liver.

While AlloPipe only focuses on HLA class I embedded peptides, both HLA class I and class II restricted mHAgs contribute to alloreactivity. Class II restricted mHAgs are indeed presented to the TCRs of CD4+ T‐lymphocytes, whose activation supports alloreactivity by synthesising cytokines that modulate the alloreactivity of CD8+ T‐lymphocytes, participating in the activation of B lymphocytes and exerting cytotoxicity via the FAS/FASL system [22]. We acknowledge that AlloPipe does not provide a prediction of class II affinity. However, intermediate tables containing comprehensive peptides list can be used to perform additional prediction using, for instance, NetMHCIIpan 4.3 [23], or MixMHC2pred [17].

Alloresponses can also occur through tolerogenic pathways, including regulatory responses, as immunogenicity depends not only on antigen presentation but also on the context of immune activation and peripheral tolerance mechanisms. Therefore, the presence of an mHAg does not necessarily lead to HvG and/or GvH effect. AMS should be considered as an indication of maximal alloreactive potential for a given donor/recipient pair of which unrelated and related pairs have different maxima. This highlights the challenges of the interpretation of the score in the context of a general population. To the best of our knowledge, AlloPipe is the first tool to provide robust and reliable calculation of (i) amino acid mismatches of interest, (ii) related peptides that are mHAgs candidates and (iii) their affinity towards HLA molecules within a few hours and with minimal computational effort, that is, in a timeframe and material setting compatible with graft allocation. The web server allogenomics.com aims to make mHAgs imputation accessible to clinicians without a bioinformatics background at the time of graft allocation to provide insights into donor selection, adjustment of immunosuppressive therapies and post‐transplant follow‐up. AlloPipe is filling the technological gap of assessing the mHAgs within a donor/recipient pair from their genomic data. Building and sharing robust and open‐source tools appears as a prerequisite, or even the first step, to sustain forthcoming clinical trials that can prospectively validate the value of the concept and the method beyond the tool. The dramatic improvement of high‐throughput sequencing technologies [24], together with better accessibility of the platforms, timing and costs, will encourage its use.

Our software is published under an MIT licence and can be downloaded at the following address: https://github.com/huguesrichard/Allopipe.

## Author Contributions

**A.D.:** conceptualization, validation, formal analysis, investigation, data curation, writing – original draft. **P.D.:** software, formal analysis, data curation **P.L.:** validation, formal analysis, software, data curation, writing – review and editing. **R.C.:** software. **A.A.:** conceptualization, methodology, resources, writing – review and editing, funding acquisition. **L.M.:** conceptualization, methodology, resources, writing – review and editing, funding acquisition. **H.R.:** conceptualization, methodology, software, formal analysis, writing – review and editing, supervision, project administration.

## Funding

A.D. was financed by a DrEAM fellowship provided by university of Lorraine (France). Part of the sequencing was financed by the “France Génomique” consortium.

## Ethics Statement

All patients gave their written consent for the generation and use of genetic data for research purposes, as well as clinical data collection.

## Conflicts of Interest

The authors declare no conflicts of interest.

## Supporting information


**Appendix 1** Allo‐count quality filters and default parameters.
Appendix 2: AMS table format.
Appendix 3: af‐AMS table format.
Supplementary data 1: Workflow Sentieon.
Supplementary data 2: Parameters for VEP annotation.
Supplementary figure 1: Distribution of AMS scores after exclusion of the three outliers' pairs.
Supplementary figure 2: Contribution of rare variants to the number of reconstructed peptides.
Supplementary figure 3: Validation of the AMS computation.

## Data Availability

The data that support the findings of this study are available on request from Alice Aarnink. The data are not publicly available due to privacy or ethical restrictions.
