# Supplementary material for: AlloPipe and Its Web Server Allogenomics: From Genomic Data to Candidate Minor Histocompatibility Antigens
Source: HLA. 2026 Feb 11;107(2):e70590. doi: 10.1111/tan.70590 (PMC12893849; doi:10.1111/tan.70590)
Supplement: Supplementary file 1 — Appendix 1 Allo‐count quality filters and default parameters. Appendix 2: AMS table format. Appendix 3: af‐AMS table format. Supplementary data 1: Workflow Sentieon. Supplementary data 2: Parameters for VEP annotation. Supplementary figure 1: Distribution of AMS scores after exclusion of the three outliers' pairs. Supplementary figure 2: Contribution of rare variants to the number of reconstructed peptides. Supplementary figure 3: Validation of the AMS computation. [file TAN-107-e70590-s001.docx]

APPENDICES

Appendix 1

Title: Quality filters with default parameters

Caption: summary of the parameters that can be provided to Allo-count for quality filtering, with theyir description and default value.

| **Parameter (per position)** | **Default** | **Meaning** |
| --- | --- | --- |
| **minimal depth** | 20 | Variant must be covered by *at least* [minimal depth] reads to be kept. |
| **maximal depth** | 400 | Variant must be covered by *at most* [maximal depth] reads to be retained. |
| **minimal allelic depth** | 5 | Allele must be covered by at least [allelic depth] reads to be retained |
| **homozygosity threshold** | 0.2 | Maximum ratio between the REF call and the ‘Depth’ for the variant. If the estimated ratio is above ‘htr’ (or below 1-’htr’), the call is not considered |
| **genotype quality** | 0 | Lowest accepted value for the predicted genotype quality. |
| **maximal indels length** | 3 | Variants longer than this value are not considered. |
| **gnomADe allele frequency threshold** | 1% | Minimal frequency of existing variant in gnomAD exomes combined pop currently ulation |

Appendix 2

Title: AMS table format

Caption: Summary of the information reported in the AMS table, with column name, type and explanation of the column.

| **VCF informations** | | |
| --- | --- | --- |
| **CHROM** | string | Chromosome of the variant |
| **POS** | int | Position on the chromosome |
| **ID_{x, y}** | string | Reference SNP cluster ID for the donor (x) or recipient (y) |
| **REF, ALT** | string | REF and ALT alleles at the given position |
| **QUAL_{x, y}** | float | Phred-scaled quality score for the assertion made in ALT |
| **FILTER_{x, y}** | string | PASS if this position has passed all filters |
| **FORMAT_{x, y}** | list | Format of the sample column post AlloPipe processing |
| **Sample_{x, y}** | string | Sample information regarding the position. Note that the column name is the one provided in the original VCF |
| **Sample information** | | |
| **GT_{x, y}** | string | Predicted genotype of the sample |
| **GQ_{x, y}** | float | Score of quality of the predicted genotype |
| **AD_{x, y}** | string | Allelic depth |
| **FT_{x, y}** | string | Sample genotype filter indicating if this genotype was “called” |
| **phased_{x, y}** | string | Predicted genotype containing phased information (if provided in the sample column) |
| **DP_{x, y}** | int | Sequencing Depth at position |
| **TYPE_{x, y}** | string | Type of genotype (homozygous, heterozygous) |
| **VEP information** | | |
| **consequences_{x,y}** | int | All the columns with a consequence with the number of times it is recorded in transcripts for the variant |
| **transcripts_{x, y}** | string | Transcripts recorded for the variant |
| **genes_{x, y}** | string | Genes recorded for the variant |
| **aa_REF, aa_ALT** | string | Amino-acid for REF and ALT alleles for the variant |
| **gnomADe_AF_{x, y}** | float | Frequency of existing variant in gnomAD exomes combined population |
| **aa_ref_indiv_{x, y}, aa_alt_indiv_{x, y}** | string | REF and ALT amino-acids recorded for the sample (x and y) |
| **aa_indiv_{x, y}** | string | REF and ALT amino-acids combined in one column |
| **AlloCount informations** | | |
| **Diff** | string | Difference between the amino-acids of both samples |
| **Mismatch** | int | Number of mismatches in the diff field |
| **Mismatch_type** | String | Type of mismatch (homozygous, heterozygous) |

Appendix 3

Title: af-AMS table format

Caption: summary of the information reported in the af-AMS table, with column name, type and explanation of the column.

| **NetMHCpan information** | | |
| --- | --- | --- |
| hla_peptides | string | Potential ligand peptide built from VEP information and Ensembl information |
| Gene_id | string | Ensembl Gene ID |
| NB | int | Number of Weak Binding/Strong Binding peptides accross given HLA |
| EL-score | float | Raw prediction score |
| EL_Rank | float | Rank of the predicted EL-score compared to a set of random natural peptides |
| BA-score | float | Binding-Affinity score |
| BA_Rank | float | Rank of the predicted BA-score |
| HLA | string | Specified MHC molecule / Allele name |
| Transcript_id | string | Ensembl Transcript ID |
| Peptide_id | string | Ensembl Peptide ID |

SUPPLEMENTARY DATA

Supplementary data 1: Workflow Sentieon®

Version: 202308.03

Model: DNAscope Illumina WES 2.1

Genome reference: GRCh38 (hs38DH)

dbSNP: v156

<https://www.biorxiv.org/content/10.1101/2022.05.20.492556v1.full.pdf>

Supplementary data 2: Parameters for VEP annotation

VEP: v113

--af_gnomade --assembly GRCh38 --cache --coding_only --pick_allele --use_given_ref --vcf

Supplementary figure 1

Title: Distribution of AMS scores after exclusion of the three outliers’ pairs

Caption: Violin plots comparing the AMS values for the cohorts that are geno-identical (left) or haplo-identical (right), after excluding three pairs considered as outliers. ** indicates a significative difference with the ANOVA test (on the AMS distribution after exclusion of the pairs).


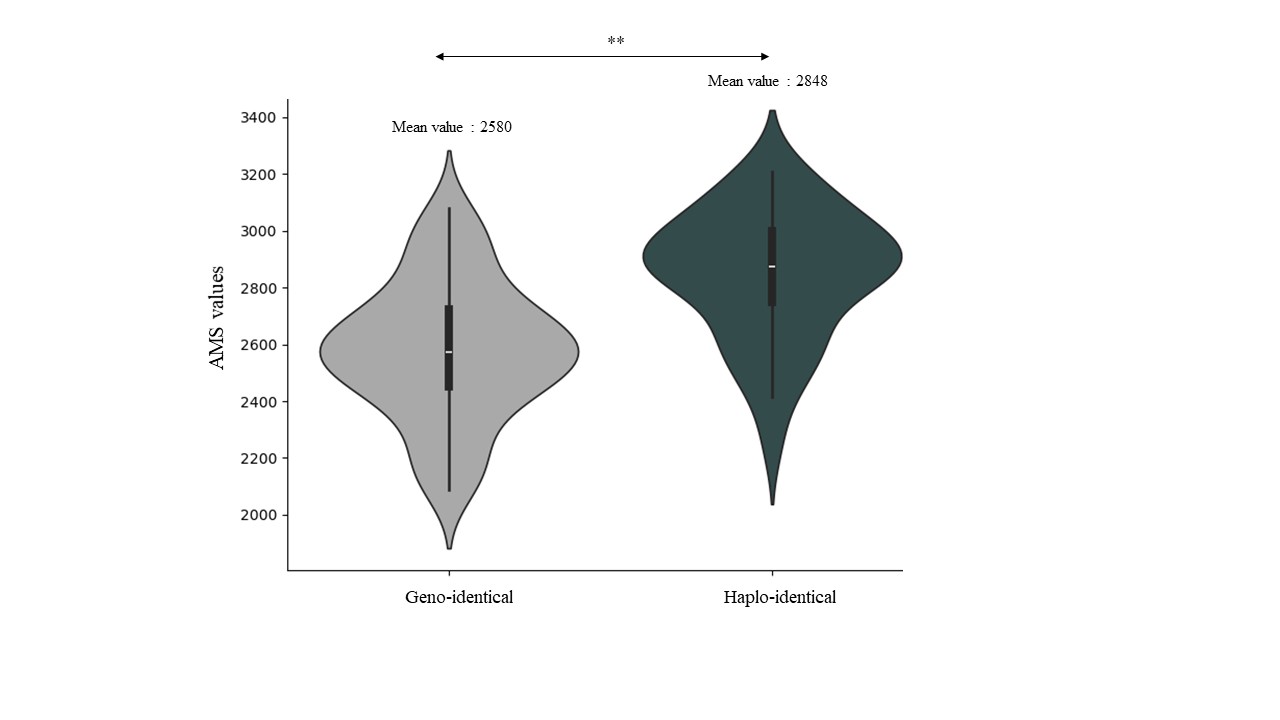


Supplementary figure 2

Title: Contribution of rare variants to the number of reconstructed peptides

Caption: Each bar represents one pair. Blue bars represent the number of peptides reconstructed after running VEP with the default parameters, i.e. gnomADe_AF threshold set at 1%. Red bars represent the number of additional peptides retrieved with the gnomADe_AF threshold set at 0%.

Supplementary figure 3

Title: Validation of the AMS computation.


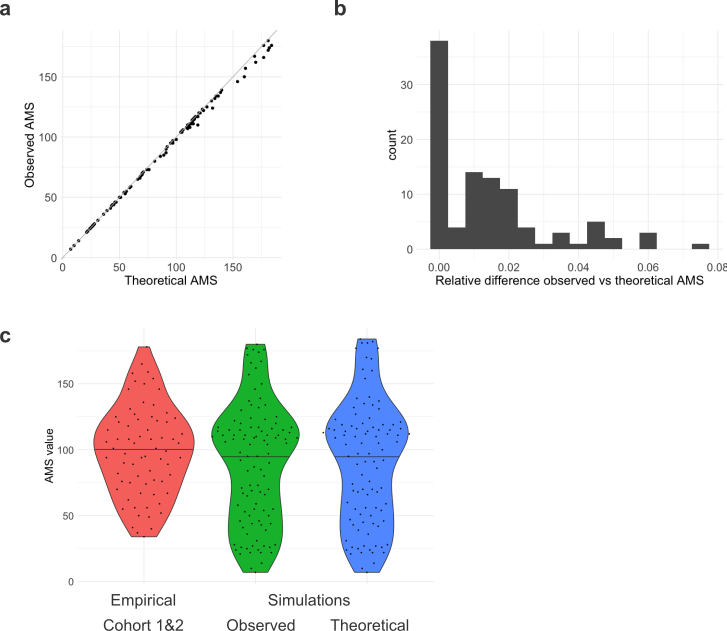


Caption: Evaluation of the AMS accuracy after simulating siblings’ genotypes from chromosome 8. a) Comparison of the theoretical value of the AMS (x axis) against the value observed after running Allo-Count on the sequencing data (y axis). The first intercept is represented by a grey line. b) Relative difference of the observed and the theoretical AMS. c) Distribution of the AMS values for the empirical data from Cohort 1 and 2 joined (red), the theoretical value from the simulation (blue) and the observed one after sequencing (green).
